# Age, gender, and voided volume dependency of peak urinary flow rate and uroflowmetry nomogram in the Indian population

**DOI:** 10.4103/0970-1591.57912

**Published:** 2009

**Authors:** Vikash Kumar, Jayesh V. Dhabalia, Girish G. Nelivigi, Mahendra S. Punia, Manav Suryavanshi

**Affiliations:** Department of Urology, KEM Hospital, Parel, Mumbai 400 012, India

**Keywords:** Flow rate nomogram, uroflowmetry, uroflow, uroflowmetry nomogram, urodynamics

## Abstract

**Objectives:**

The objective of this study was measurement of urine flow parameters by a non invasive urodynamic test. Variation of flow rates based on voided volume, age, and gender are described. Different nomograms are available for different populations and racial differences of urethral physiology are described. Currently, there has been no study from the Indian population on uroflow parameters. So the purpose of this study was to establish normal reference ranges of maximum and average flow rates, to see the influence of age, gender, and voided volume on flow rates, and to chart these values in the form of a nomogram.

**Methods:**

We evaluated 1,011 uroflowmetry tests in different age groups in a healthy population (healthy relatives of our patients) 16-50 year old males, >50 year old males, 5-15 year old children, and >15 year pre-menopausal and post-menopausal females. The uroflowmetry was done using the gravitimetric method. Flow chart parameters were analyzed and statistical calculations were used for drawing uroflow nomograms.

**Results:**

Qmax values in adult males were significantly higher than in the elderly and Qmax values in young females were significantly higher than in young males. Qmax values in males increased with age until 15 years old; followed by a slow decline until reaching 50 years old followed by a rapid decline after 50 years old even after correcting voided volume. Qmax values in females increased with age until they reached age 15 followed by decline in flow rate until a pre-menopausal age followed by no significant decline in post-menopausal females. Qmax values increased with voided volume until 700 cc followed by a plateau and decline.

**Conclusions:**

Qmax values more significantly correlated with age and voided volume than Qavg. Nomograms were drawn in centile form to provide normal reference ranges. Qmax values in our population were lower than described in literature. Patients with voided volume up to 50 ml could be evaluated with a nomogram.

## INTRODUCTION

Uroflowmetry is the measurement of the rate of urine flow over time. The measurement of urine flow is non invasive and is the easiest uro-dynamic test useful as preliminary or follow-up investigation of the lower urinary tract symptom. The clinical usefulness of uroflow rate has been attenuated by the lack of absolute values defining normal limits.[1] Urinary flow rates depend on voided volume in a nonlinear fashion.[2–5] Nomograms are required to see the change in flow rates at different voided volumes, and the use of nomograms overcome the danger of referencing flow rates to any one voided volume. Nomograms in centile form were constructed to provide normal reference ranges in various age groups for urinary flow rates covering a wide range of voided volumes.[2–4,6–10] Siroky, *et al*. were among the first to develop a nomogram that allowed uroflow to be corrected for voiding volume.[2] There are racial differences described in African and Caucasian women for urodynamic parameters.[11] However, there is no study for the Indian population on uroflowmetry.

The purpose of this study was to establish normal reference ranges for urinary flow rates over a wide range of voided volumes, to see the influence of age, gender, and voided volume on flow rates, and to chart these values in the form of nomogram charts for the standard Indian population.

## MATERIALS AND METHODS

A prospective study was conducted by the Department of Urology, K.E.M Hospital in Mumbai from August 2005 to November 2007. Healthy relatives of our admitted patients were recruited after taking valid consent. Different age groups of the healthy population were: 16 to 50 year males (Group I), > 50 year old males (Group II), 5 to 15 year olds (Group III), >15 year old pre-menopausal females (Group IV), and post-menopausal females (Group V). These five groups were analyzed separately and intergroup differences were statistically analyzed.

We used the gravitimetric method for uroflowmetry, using Santron 2pl; PC-based urofloweter. Calibration was initially performed using the internal self calibration program on the apparatus and repeated at intervals to ensure consistency.[12] The checking of voided volume, flow time, and average flow rate was performed using known fluid volumes and time (stopwatch). We descriptively analyzed flow chart parameters and used statistical calculation for drawing uroflow nomograms. Nomograms were constructed depicting the normal Gaussian distribution of maximum and average urinary flow rate at volumes ranging from 50 to 1000 cc. These healthy populations were evaluated by history, examination, and investigations. Evaluated parameters were voided volume, maximum flow rate, average flow rate, flow time, and time to Qmax.

Exclusion criteria were patients with urological complaints or a history of neurological disorders. Other healthy volunteers were included in the study.

### Statistical Analysis

Statistical analysis was performed using SPSS software (SPSS, Inc.). Several transformations of data were assessed and the goodness-of-fit tested to determine whether a linear, hyperbolic, parabolic, or logarithmic function best described the relationship between the maximum or average flow rates and the voided volume. The Quintile regression method was used to establish the percentile levels (5, 10, 25, 50, 75, 90, and 95). Nomograms were presented in centile form and prepared for each group. The difference was assessed for significance using a student's t test. Data was analyzed for “goodness of fit” using the Kolmogorov Smirnoff test (K-S-test).

## RESULTS

We evaluated uroflowmetry data of 1,011 patients from a healthy population; 262 patients were in Group I (16-50 year old males), 239 patients were in Group II (> 50 year old males), 217 patients were in Group III (children 7-14 years old), 207 patients were in Group IV (pre-menopausal females), and 92 patients were in Group V (post-menopausal females).

In Group I, the median age was 31 years old. The mean voided volume was 440 ± 215 ml. The mean maximum flow rate and average flow rate were 22.5 ± 9.2 ml/sec and 13.05 ± 6.12 ml/sec, respectively. The correlation between Qmax and Qavg with voided volume were significant (Pearson's correlation coefficient r = 0.413 and r = 0.488, p< 0.01, respectively). The higher the voided volume, the higher the flow rates. Qmax values showed a significant correlation with age (r: -0.15, P = 0.012). Qavg is not significantly correlated with age, (Pearson's correlation r: -0.11, *P* value = 0.08). There was a decline in Qmax by 1 ml/sec/decade.

Equation for the flow rates based on voided volume and age in Group I

√ Maximum flow rate (Qmax) = 3.58 + 0.482√ (VV) − 0.145× (age)

**Figure 1:**
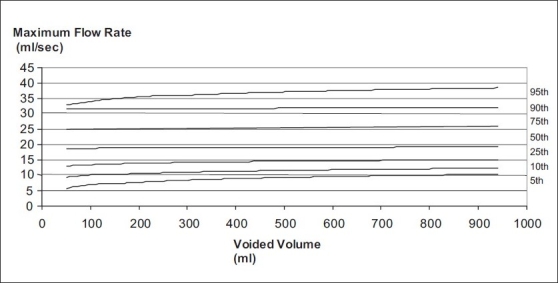
Uroflowmetry nomogram for maximum flow rates in the male population 16-50 years old in India

Nomogram charts for maximum flow rate and voided volume were prepared and plotted based on regression analysis [Figure 1].

The median age in Group II was 67 years old. Voided volumes were 297.78 ± 142 ml. The mean maximum flow rate and average flow rate were 17 ± 7.16 ml/sec and 8.9 ± 4.06 ml/sec. Qmax and Qavg are well correlated with voided volume (r: 0.576, *P* value: 0.001 and r: 0.519, *P* value-.0001). Qmax and Qavg in this group are negatively correlated with age (r: - 0.297, *P* value - 0.001. and r:-4.00, *P* value – 0.0001).

Equation for the flow rates based on voided volume and age in Group II

√Maximum flow rate = 3.2 + 0.544 √ (VV) − 0.154(age)

**Figure 2:**
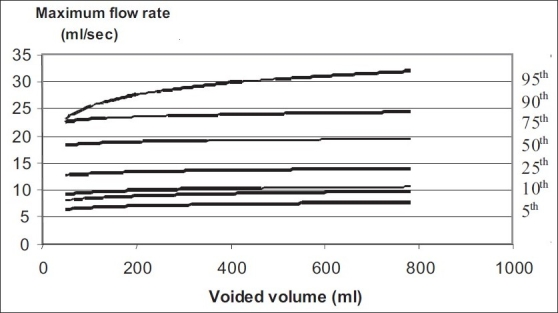
Uroflowmetry nomogram for maximum flow rates in > 50 year old male population in India

Nomogram charts for maximum flow rate and voided volume are shown in Figure 2.

A comparison between Groups I and II and the statistical differences are discussed in Table 1.

**Table 1:** Comparison of various flow parameters in between Group I (16-50 years old) and Group II (>50 years old) male

Parameters evaluated	Group I (16-50 year) males (n = 262)	Group II (>50 year) males (n =239)	P-value (Student t test)
Maximum flow rate(ml/sec)(Mean ± S.D)	22.5 ± 9.2	17 ± 7.16	0.001
Average flow rate(ml/sec) (Mean ± S.D)	13.05 ± 6.12	8.9 ± 4.06	0.001
Voiding time(sec) (Mean ± S.D)	37 ± 19.33	37.68 ± 19.08	0.619
Time to Q max (Mean ± S.D)	8.49 ± 3.82	10.94 ± 9.28	0.001

Out of 299 females, 202 were pre-menopausal (Group IV) and 97 were post-menopausal (Group V). The two groups were analyzed separately to assess the effect of hormonal withdrawal with menopause on the physiology of the urethra and pelvic floor and the effect on uroflow parameters. The median age in the pre-menopausal group was 32. The mean voided volume was 399 ± 189 ml. The mean maximum flow rate and average flow rate were 21.8 ± 8.22 ml/sec and 12 ± 4.6 ml/sec, respectively. Qmax values were negatively correlated with age (Pearsons correlation r: - 0.222, *P* value<0.05).

Equations for the flow rates based on voided volume an age in Group IV

√Maximum flow rate = 4.207 + 0.470 √ (VV) − 0.174(age)

In the group of post-menopausal females, the median age was 61 years old. The mean voided volume was 362 ± 141 ml. The mean maximum flow rate and average flow rate were 17.59 ± 5.59 ml/sec and 10.2 ± 3.52 ml/sec, respectively. Qmax values were negatively correlated with age, but they were statistically non-significant (r: 0.036, *P* value =.08).

Equations for the flow rates based on voided volume and age in Group V

√Maximum flow rate = 1.36 + 0.575 √ (VV) − 0.086(age)

**Figure 3:**
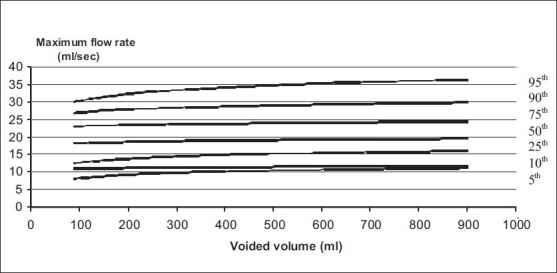
Uroflowmetry nomogram for maximum flow rates in premenopausal female in India

**Figure 4:**
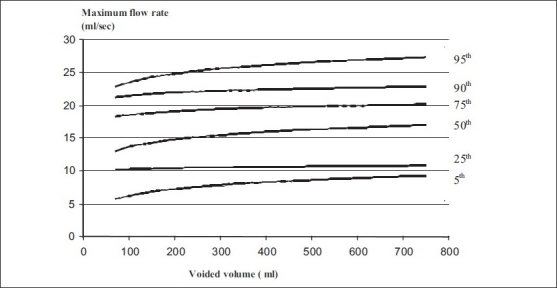
Uroflowmetry nomogram for maximum flow rates in postmenopausal females in India

Nomograms for pre-menopausal and post-menopausal females are shown in Figures 3 and 4 and a comparison of pre-menopausal and post-menopausal females is discussed in Table 2.

**Table 2:** Comparison of various flow parameters in between Group IV (Premenopausal female) and Group V (Postmenopausal female)

Parameters evaluated	Group IV (premenopausal females) n = 207	Group V (post-menopausal females) n =92	P-value (Student t test)
Maximum flow rate (Mean ± S.D)	21.8±8.22	17.59±5.59	0.0001
Average flow rate (Mean ± S.D)	12 ± 4.6	10.2±3.52	0.0009
Voiding time (Mean ± S.D)	35.66 ± 16.51	41 ± 19.5	<0.0153
Time to Q max (Mean ± S.D)	8.03 ± 3.85	10± 6.17	0.09

The flow rate was not statistically different in nulliparous or multiparous females.

Group III consisted of 5-15 year old children; the median age was 9 years old. The mean voided volume was 220 ± 135 ml. The mean maximum flow rate and average flow rate was 17.7 ± 6.2 ml/sec and 10.08 ± 3.44 ml/sec, respectively. Qmax and Qavg positively correlated with age (r = 0.702, *P* value = 0.0001 and r = 0.752, *P* value = 0.0001). Qmax values and voided volume was highly correlated (r 0.662, *P* value: 0.0001).

√ Maximum flow rate: 1.996+0.397 √ (vv) + 0.485(age)

On analyzing girls and boys separately, for girls the mean age was 10.05 years old and voided volume was 236.4 ± 92 ml. Qmax and Qavg values were 19.33 ± 6.34 ml/sec and 12.3 ± 2.56 ml/sec. For boys, the mean age was 9.4 years old and the mean voided volumes were 222 ± 112 ml/sec. Mean Qmax and Qavg values were 16.5 ± 5.72 ml/sec and 10.5 ± 3.75 ml/sec, respectively.

**Figure 5:**
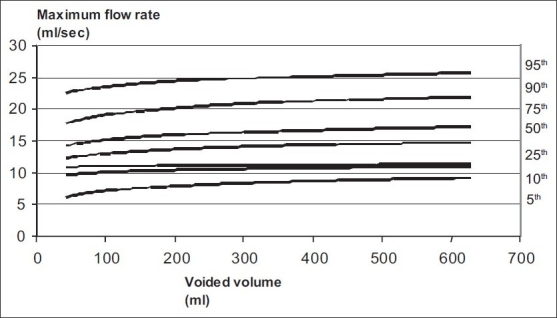
Uroflowmetry nomogram for maximum flow rates in males 5-14 years old in India

**Figure 6:**
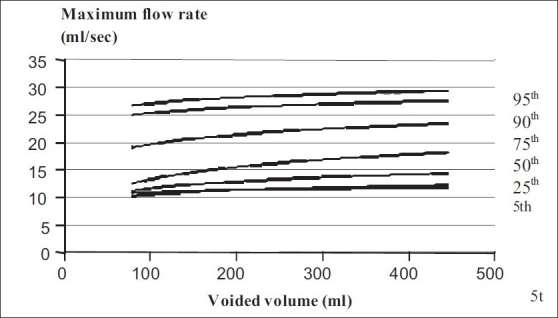
Uroflowmetry nomogram for maximum flow rates in females 5-14 years old in India

The maximum flow rate nomogram for boys and girls is shown in Figures 5 and 6 and a comparison of the boys and girls in Group III are discussed in Table 3.

**Table 3:** Comparison of various flow parameters among Group III (males and females)

Parameters evaluated	Boys (7-14 years old) n = 124	Girls (7-14 years old) n = 93	P-value (Student t test)
Maximum flow rate (Mean ± S.D)	16.5 ±5.72	19.33±6.34	0.005
Average flow rate (Mean ± S.D)	9.6 ±3.56	11.25±2.83	0.001
Voiding time (Mean ± S.D)	20 ± 8.93	12.3 ± 2.56	<0.001
Time to Q max (Mean ± S.D)	8.6 ± 5.2	7.9 ± 4.27	0.2931

## DISCUSSION

Mean maximum and average flow rate parameters in different age groups are shown in Table 4. Nomograms were constructed to provide normal reference ranges for both genders for urinary flow rates covering a wide range of voided volumes and in centile form. The use of statistical transformations in their construction overcame the problems created by inaccuracies when untransformed standard deviations were used i.e., the Siroky nomogram.[2,13]

**Table 4:** Mean maximum and average flow rate parameters in different age group

**Male population**	**Maximum flow rate**	**Average flow rate**

16-50 years old	22.8 ml/sec	13.22ml/sec
>50 year	17.04 ml/sec	8.9ml/sec

**Female population**	**Maximum flow rate**	**Average flow rate**

Pre-menopausal	21.8 ml/sec	12 ml/sec
Post-menopausal	17.59 ml/sec	10.2 ml/sec

**Pediatric population**	**Maximum flow rate**	**Average flow rate**

Girls	19.33 ml/sec	11.25 ml/sec
Boys	16.9 ml/sec	9.6 ml/sec

Among the male population, Qmax values in the 16 to 50 year old group were 22.8 ± 9.33 ml/sec - significantly higher than in the > 50 year old and 5 to 15 year old groups, which were 17.04 ± 7.1 ml/sec and 16.9 ± 5.38 ml/sec, respectively. Similar statistical differences were found by Suebnukanwattana, *et al*. in his study (2003) comparing two groups. Group I comprised of 50 males, aged 18-30 years old and Group II comprised of 20 males pre-elderly, aged 50-60 years old. Qmax values in our population of adult males was 22.8 ml/sec, which is lower than 28.4 ml/sec and 31.2 ml/sec in the Austrian male adolescent and Thai population, respectively.[14,15] Qmax values in the elderly population were 17.04 ml/sec significantly lower than the study in the Thai population (27.5 ml/sec).[15]

Among female groups, the Qmax values were 22.98 ml/sec in the pre-menopausal group and 19.04 ml/sec in the post-menopausal group. There was a negative correlation with age and Qmax and Qavg. Our finding is different from Haylen, *et al*., who reported no dependence of flow rate with age. No correlation could be found with Qmax and parity. A similar relation has also been confirmed by the reports of Haylen, *et al*.[4]

A significant positive correlation with age was seen in the 5 to 15 year old age group and a negative correlation of Qmax values with age was seen in the 15 to 50 year old group and in the more than 50 year old group. Similar negative correlations with age were shown by the Liverpool nomogram and the study in the Thai population.[4,15] We found a strong relationship between Qmax and Qavg values with voided volume in all the three groups. A similar strong correlation was found by Siroky, *et al*. and Haylen, *et al*.[2,4] Uroflowmetry nomograms were drawn based on these positive correlations between voided volume and flow rates.

On comparing the flow rates of the male and female groups, Qmax values were 22.8 ± 9.33 ml/sec and 20.53 ± 7.75ml/sec, respectively. There was no statistically difference found between the two groups. Voided volume constant maximum flow rates were higher in females. Similarly, Drach, *et al*. reported in their study that normal female subjects have a higher Qmax value for a given voided volume than do normal males of the same age.[16,17,18] Normally, females have a short urethra usually with minimal outlet resistance. Voiding time was prolonged in post-menopausal females, which is significantly higher than the pre-menopausal and 5 to 15 year old groups.

Among the pediatric population, flow rates for girls and boys were 19.2 ± 6.95 ml/sec and 16.9 ±5.38 ml/sec, respectively (p<0.001). Similar results have been seen by Guitierrez Segura and Kajbafzadeh, *et al*.[19,20] Guitierrez Segura's report confirmed that the Qmax value increased with volume and age.[19] Mean values were higher in girls than in boys. In our study, we also found a positive correlation of age with maximum flow rates. On comparing flow time and time to Qmax, there is no statistically significant difference in both groups for time to Qmax but there was significant difference between the two groups in voiding time (*P* value<0.001). Guitierrez Segura's and Jensen, *et al*. show that flow time is shorter in girls.[19]

Qmax is positively correlated with voided volume that is seen up to 700 ml voided volume; after 700 ml there is a plateau followed by a decline. A similar report was conducted by Dominik, *et al*., who found a positive correlation until voided volumes of 350 ml in the adolescent population, but constant until 500 ml and a decrease in Qmax values after 500 ml.[21,22]

Voided volume changes significantly with age in the 5 to 15 year old group. To eliminate the factor of rising voided volume on rising flow rate with age, an analysis of flow rate at a constant voided volume was done. At a constant voided volume of 200 to 250 ml, the maximum flow rate is significantly correlated with age.

## CONCLUSIONS

Maximum flow rate (Qmax) is more significantly correlated with age and voided volume than average flow rate (Qavg); hence, Qmax is the single most useful parameter of uroflowmetry. Qmax increases with age in the pediatric population and decreases with age in the adult and elderly population. Qmax in girls was significantly higher than in boys. No artificial restriction of voided volume e.g., minimum 150 ml, is appropriate. Patients with voided volume up to 50 ml can also be evaluated with the help of a nomogram.

Qmax values in the adult population (15-50 years old) and the elderly population were lower than in the Thai and Austrian healthy population. Nomograms in centile form were constructed to provide normal reference ranges for urinary flow rates and covered a wide range of voided volumes.
